# Screening and Identification of Lujo Virus Inhibitors Using a Recombinant Reporter Virus Platform

**DOI:** 10.3390/v13071255

**Published:** 2021-06-28

**Authors:** Stephen R. Welch, Jessica R. Spengler, Sarah C. Genzer, Payel Chatterjee, Mike Flint, Éric Bergeron, Joel M. Montgomery, Stuart T. Nichol, César G. Albariño, Christina F. Spiropoulou

**Affiliations:** Viral Special Pathogens Branch, Centers for Disease Control and Prevention, U.S. Department of Health and Human Services, Atlanta, GA 30329, USA; yos6@cdc.gov (S.R.W.); wsk7@cdc.gov (J.R.S.); muz8@cdc.gov (S.C.G.); mwe9@cdc.gov (P.C.); vfa3@cdc.gov (M.F.); exj8@cdc.gov (É.B.); ztq9@cdc.gov (J.M.M.); stn1@cdc.gov (S.T.N.); bwu4@cdc.gov (C.G.A.)

**Keywords:** Lujo virus, viral hemorrhagic fever, arenavirus, reporter virus, reverse genetics, antiviral screen, therapeutic, emerging viruses

## Abstract

Lujo virus (LUJV), a highly pathogenic arenavirus, was first identified in 2008 in Zambia. To aid the identification of effective therapeutics for LUJV, we developed a recombinant reporter virus system, confirming reporter LUJV comparability with wild-type virus and its utility in high-throughput antiviral screening assays. Using this system, we evaluated compounds with known and unknown efficacy against related arenaviruses, with the aim of identifying LUJV-specific and potential new pan-arenavirus antivirals. We identified six compounds demonstrating robust anti-LUJV activity, including several compounds with previously reported activity against other arenaviruses. These data provide critical evidence for developing broad-spectrum antivirals against high-consequence arenaviruses.

## 1. Introduction

Lujo virus (LUJV), the first highly pathogenic arenavirus identified in Africa in over 40 years, was recognized after a cluster of severe viral hemorrhagic fever (VHF) cases in Southern Africa in September and October of 2008 [1,2]. The index case was identified in Zambia, and four subsequent nosocomial cases were detected in associated healthcare workers in South Africa. The infections initially presented as a non-specific febrile illness, but progressed in severity over 10–13 days to respiratory distress, neurological signs, and circulatory collapse [1]. To date, no additional cases of LUJV infection have been reported. However, the lack of a defined etiology or source of exposure for the index case; the apparent ease with which primary, secondary, and tertiary contacts were infected; and the unusually high case fatality rate (80%) caused significant alarm.

The family Arenaviridae encompasses three genera: *Mammarenavirus*, *Reptarenavirus*, and *Hartmanivirus* [3]. With the exception of the bat-borne Tacaribe virus, rodents are the natural hosts of the mammalian-specific mammarenaviruses, with each viral species closely associated with a single rodent species reservoir [4,5]. Infected rodents develop a persistent infection and continually shed the virus in body secretions such as urine and feces; human transmission occurs via contact with these secretions, either directly or in contaminated food products [6]. Mammarenaviruses are divided into two groups based on the geographic, genetic, and epidemiological relationship with their respective rodent hosts: Old World (or lymphocyte choriomeningitis/Lassa complex) species and New World (or Tacaribe complex) species [7]. Several *Mammarenavirus* species in both these groups can cause severe VHF disease in humans. Highly pathogenic Old World complex viruses such as LUJV and Lassa virus (LASV) are found in Africa, whereas the New World pathogens such as Junin (JUNV), Guanarito (GTOV), Machupo, and Chapare viruses are found in South America [8]. Treatment options for these highly pathogenic mammarenaviruses remain limited. The only therapeutic agent currently in clinical use is convalescent plasma therapy for JUNV patients [9], although broad-spectrum antiviral nucleoside analogs such as ribavirin and favirpiravir (T-705) have demonstrated some clinical success in treating LASV if treatment is started early and when clinical signs are still mild [10]. While currently no FDA-approved drugs are licensed to specifically treat these pathogens, advances in drug discovery targeting specific areas of the viral life cycle, such as entry, trafficking, RNA replication, viral and host cell protein interactions, and budding are uncovering several promising avenues of research [11].

Here, we describe the development and rescue of a novel recombinant ZsGreen1 (ZsG)-expressing reporter LUJV, termed rLUJV/ZsG. By incorporating rLUJV/ZsG into a high-throughput antiviral compound screening assay, we were able to rapidly evaluate potential therapeutic candidates that were effective against LUJV. A panel of compound candidates was selected based on: (1) demonstrated activity against other arenaviruses; (2) demonstrated activity against other negative-sense RNA viruses; and (3) preliminary data from large in-house screening assays against multiple virus species. Of the 83 compounds tested, 20 demonstrated robust anti-LUJV activity; 6 were further confirmed to potently inhibit wild-type (wt) LUJV infection. Using LUJV in these assays provides critical support for antiviral activity of previously identified and novel compounds against re-emerging and emerging highly pathogenic arenaviruses.

## 2. Materials and Methods

### 2.1. Biosafety

All work with the infectious virus was conducted in a biosafety level 4 (BSL-4) laboratory at the Centers for Disease Control and Prevention (CDC, Atlanta, GA, USA) following established BSL-4 standard operating procedures approved by the Institutional Biosafety Committee. All recombinant virus work was approved by the Centers for Disease Control and Prevention Institutional Biosafety Committee.

### 2.2. Cells

Vero-E6, Huh7 (from Apath LLC, New York, NY, USA), and BSR-T7/5 cells were cultured in Dulbecco’s modified Eagle’s medium supplemented with 5% (*v*/*v*) fetal calf serum, non-essential amino acids, 100 U/mL penicillin, and 100 μg/mL streptomycin.

### 2.3. Rescue of Recombinant LUJV

Recombinant reporter LUJV expressing the green fluorescent protein ZsGreen1 (rLUJV/ZsG) was generated using a previously described reverse genetics system [12]. Briefly, to rescue recombinant LUJV, T7 promoter-based plasmids containing full-length antigenomic sense LUJV L and S genome segments (prototypic Zambian strain; Genbank NC_012777.1 and NC_012776.1, respectively) were transfected into BSR-T7/5 cells. The reporter rLUJV/ZsG was generated by substituting the full-length antigenomic sense S segment with a modified version encoding ZsG. At 5 days post-transfection, cell culture supernatants were harvested, clarified by low-speed centrifugation, and used to infect Vero-E6 cells. Virus stocks harvested 3–4 days post-infection were quantified in Vero-E6 cells by tissue culture infective dose 50 (TCID_50_) assays, using either immunofluorescence (wt virus) or ZsG expression (reporter virus) [13].

### 2.4. Next-Generation Sequencing and Bioinformatics

RNA was extracted from cell culture supernatants (clarified by low-speed centrifugation) using the MagMAX-96 Total RNA Isolation Kit (Thermo Fisher Scientific, Waltham, MA, USA) on a 96-well Roche MagMAX extraction platform with a DNase-I treatment step according to manufacturer’s instructions. RNA sequencing was performed using KAPA RNA HyperPrep kit (Roche, Basel, Switzerland) for library preparation and analyzed on the MiniSeq system (Illumina, San Diego, CA, USA).

### 2.5. Antiviral Compound Screening

Compounds were obtained from Selleckchem (Pittsburgh, PA, USA), Tocris (Bristol, UK), or MedChemExpress (Monmouth Junction, NJ, USA), with the exception of favipiravir, which was obtained from BOC Sciences (Shirley, NJ, USA). Huh7 cells were seeded at 1 × 10^4^ cells/well in a 96-well plate 16–20 h prior to treatment with compounds diluted in dimethyl sulfoxide (DMSO; final DMSO concentration 0.5%). For experiments measuring fluorescence reduction, 1 h post-treatment, cells were infected with rLUJV/ZsG (MOI 0.1; passage 1 stock from Vero-E6 cells); 72 h post-infection (hpi), ZsG fluorescence was determined using a BioTek Synergy reader (height 6 mm; 100 gain/sensitivity). For experiments measuring the reduction in wt virus titers, 1 h post-treatment, cells were infected with LUJV (MOI 0.1; prototypic Zambian strain; Genbank NC_012777.1 and NC_012776.1; passaged twice in Vero-E6 cells from original isolate); 72 hpi, supernatants were collected and viral titers determined by TCID_50_ in Vero-E6 cells. Cell viability (ATP content) was determined in parallel in compound-treated, mock-infected cells using CellTiter-Glo 2.0 (Promega, Madison, WI, USA). All experiments were performed in quadruplicate and repeated at least three times.

### 2.6. Microscopy

ZsG fluorescence in infected cells was imaged directly using an EVOS digital inverted microscope (Thermo-Fisher, Waltham, MA, USA). For immunofluorescence, cells were fixed in 10% formalin for 30 min, permeabilized in phosphate-buffered saline (PBS) containing 0.1% Triton-X100 (*v*/*v*) for 10 min, and blocked with PBS with 5% bovine serum albumin (*v*/*v*) for 30 min. Primary antibodies were α-LUJV HMAF (generated in-house, CDC, Atlanta, GA, USA, ref# 703712), and secondary antibodies were labeled with AlexaFluor488 (Thermo-Fisher, Waltham, MA, USA, #A11034).

### 2.7. Data Analysis

ZsG fluorescence values for compound-treated infected cells were normalized to mock-treated (DMSO only), infected cells and used to fit a 4-parameter equation to semi-log plots of the concentration-response data. From this, the compound concentrations inhibiting ZsG expression by 50% (EC_50_) were interpolated. Cell viability was similarly calculated in compound-treated, mock-infected cells to determine the 50% cell cytotoxicity concentration (CC_50_) of each compound. The selectivity index was calculated by dividing CC_50_ by EC_50_. Data analysis was performed using GraphPad Prism v9 (GraphPad, San Diego, CA, USA). Suitability for high-throughput screening was determined using the Z prime (Z′) score, a measure of statistical effect size, calculated as Z′ = 1 − (3 × (σ_P_ + σ_N_)/|µ_P_ − µ_N_|, where σ_P_ = standard deviation of positive control, σ_N_ = standard deviation of negative control, µ_P_ = mean of positive control, and µ_N_ = mean of negative control. Z′ values between 0.5 and 1.0 were considered acceptable. Signal-to-noise ratio was determined as (µ_P_ − µ_B_)/σ_B_, where µ_P_ = mean signal of positive control, µ_B_ = mean background signal, and σ_B_ = standard deviation of background signal. Significance was calculated using a one-sample *t*-test.

## 3. Results

### 3.1. Generation of a Recombinant LUJV Expressing ZsGreen1 Fluorescent Protein

**Figure 1:**
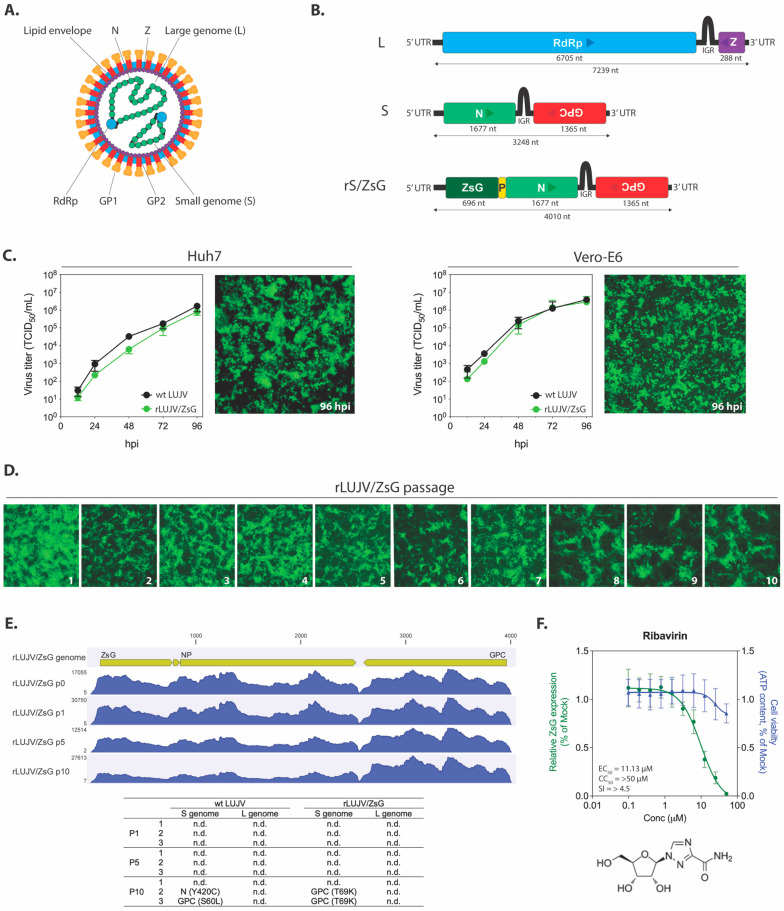
Design and characterization of rLUJV/ZsG. (**A**) Schematic representation of the Lujo virus (LUJV) virion; and (**B**) the LUJV large (L) and small (S) genome segments (antigenomic sense). Boxes represent the viral protein coding sequences (CDS) for RNA-dependent RNA polymerase (RdRp), Z matrix protein (Z), glycoprotein precursor (GPC), and nucleoprotein (N). Arrows represent the coding direction. The recombinant S genome segment (rS/ZsG) contains the ZsGreen1 (ZsG) CDS attached to the N CDS via a porcine teshovirus-1 2A (P2A; P in the figure) nucleotide sequence. GP1, glycoprotein 1; GP2, glycoprotein 2; UTR, untranslated region; IGR, intergenic region; nt, nucleotides. (**C**) Viral growth curves in Vero-E6 and Huh7 cells infected with either wild-type (wt) LUJV or rLUJV/ZsG at MOI 0.1. TCID_50_ titers were determined at 12, 24, 48, 72, and 96 h post-infection (hpi). Representative fluorescent microscopy images (10× magnification) of rLUJV/ZsG-infected cell monolayers at 96 hpi are presented. (**D**) rLUJV/ZsG was passaged 10 times in Vero-E6 cells, with representative fluorescent microscopy images presented (10 × magnification) of infected monolayers taken at time of passage (3–4 days post-infection). (**E**) NGS analysis of wt LUJV and rLUJV/ZsG passaged 10 times in Vero-E6 cells. Viral RNA analyzed at passages 1, 5, and 10 showed no changes in S genome segment coverage over the passages. The associated table indicates the non-synonymous mutations detected in the S genome segments of each of the 3 replicates at passages 1, 5, and 10 (n.d., none detected). (**F**) The rLUJV/ZsG fluorescence reduction screening assay was evaluated with the antiviral compound ribavirin. Reduction in ZsG fluorescence in Huh7 cells treated with a range of ribavirin concentrations 1 h prior to infection with rLUJV/ZsG at MOI 0.1 was determined, with fluorescence values normalized to mock-treated cells (DMSO only). Cell viability was assessed at each ribavirin concentration by measuring ATP content (green), and values were normalized to those of mock-treated cells. Each point represents the mean of quadruplicate wells, with error bars indicating standard deviation; graphs are representative of 2 independent experiments. Associated EC_50_, CC_50_, and SI values are provided, along with the chemical structure of ribavirin.

LUJV contains two ambisense genome segments termed large (L) and small (S) with respect to their relative nucleotide length (Figure 1A). Each genome segment encodes two genes: RNA-dependent RNA polymerase (RDRP) and Z protein on the L, and the nucleoprotein (N) and glycoprotein precursor (GPC) on the S (Figure 1B). Transcription of monocistronic viral mRNA is initiated at the untranslated region (UTR) and terminates at the intergenomic region (IGR).

Previous attempts to generate mammarenaviruses expressing reporter plasmids have either incorporated additional genome segments, as done with tri-segmented recombinant lymphocytic choriomeningitis virus (LCMV) [14], or introduced additional coding sequences (CDS) into the S genome segment, as in a recombinant reporter LASV [15,16]. Here, to generate a recombinant LUJV expressing the fluorescent protein ZsG, we constructed a modified S genome segment in which the ZsG coding sequence was inserted upstream of the N coding sequence, with a porcine teshovirus-1 2A peptide linker sequence (P2A) inserted between ZsG and NP (Figure 1B). This genetic format transcribes a single viral mRNA, but the two separate proteins are expressed via a ribosomal skipping event during P2A translation [17]. The modified S genome segment was incorporated into the previously described reverse genetics system for LUJV [12]. The recombinant reporter LUJV, able to express all parental viral proteins alongside the inserted ZsG reporter, was successfully rescued using this system and was termed rLUJV/ZsG.

To establish the suitability of rLUJV/ZsG as a surrogate for wt LUJV, we first investigated viral growth kinetics. Huh7 or Vero-E6 cells were infected with either wt LUJV or rLUJV/ZsG at MOI 0.1, and titers were determined at 12, 24, 48, 72, and 96 hpi. In both cell lines, growth kinetics of wt LUJV and rLUJV/ZsG were comparable at each time point assessed. In cells infected with rLUJV/ZsG, strong ZsG expression was observed in monolayers of both cell types at 96 hpi (Figure 1C). To assess the stability of the ZsG insert, we passaged rLUJV/ZsG 10 times in Vero-E6 cells (in triplicate). At each passage point, strong ZsG expression was detected in the monolayer, indicating that the ZsG-P2A-NP modification in the S genome segment was stable (Figure 1D). To further assess the genome stability, we used next-generation sequencing (NGS) to analyze the viral RNA from passages 1, 5, and 10, and compared the sequences to those of wt LUJV passaged concurrently (Figure 1E). The ZsG CDS was retained over 10 passages and no mutations were seen in any of the replicates. Similarly, no mutations were detected in the L genome segment over 10 passages. In the S genome segment, no mutations were seen over 5 passages in either wt LUJV or rLUJV/ZsG. At passage 10, 2 of the 3 replicate samples of both viruses had accumulated one non-synonymous, non-identical mutation.

### 3.2. Suitability of the LUJV Reporter Virus For Use in an In Vitro Screening Assay

To determine the suitability of rLUJV/ZsG for in vitro screening assays, potential LUJV inhibitors were screened using rLUJV/ZsG in Huh7 cells, which have been previously used in the discovery and evaluation of antiviral compounds for other VHF pathogens [16,18,19]. The assays were optimized using ribavirin, a well-documented broad-spectrum antiviral. The Z′ score (0.89) and signal-to-noise ratio (190:1) were calculated using 50 µM ribavirin as the positive control and DMSO vehicle only as the negative control and indicated a robust assay. Ribavirin dose-response curves demonstrated a concentration-dependent reduction in ZsG fluorescence, with a calculated EC_50_ value of 11.13 ± 1.4 µM and CC_50_ of >50 µM (Figure 1F). These values are broadly similar to those reported for other arenaviruses, including LASV (2.47 µM) and JUNV (18.3 µM) [16,20], indicating that rLUJV/ZsG is suitable for a screening assay that uses reduction in ZsG fluorescence to indicate inhibition of viral replication.

### 3.3. Antiviral Compound Screening

To identify anti-LUJV therapeutics, a wide range of compounds was identified from published data on arenavirus inhibitors, broad-spectrum antivirals, and preliminary in-house screens using an FDA-approved library of compounds. Virus inhibition was initially determined for each of 83 candidate compounds at three concentrations (5000, 500, and 50 nM), with cell viability confirmed concurrently (Supplementary Table S1). Twenty compounds demonstrated robust anti-LUJV activity (ZsG fluorescence reduced to <30% of control) while maintaining >80% cell viability. These compounds were further evaluated using an extended concentration curve (Table 1). Six of the twenty compounds had SI ≥ 10, demonstrating promising efficacy and applicability for therapeutic use; efficacy was further confirmed against wt LUJV.

The 20 compounds demonstrating robust anti-LUJV activity can be broadly grouped based on known or predicted modes of antiviral action as: (1) inhibitors of viral replication; (2) inhibitors of viral entry, intracellular trafficking, or virus egress; (3) protein kinase inhibitors; (4) selective estrogen receptor modulators (SERMs); or (5) miscellaneous compounds not falling into the four previous groups.

#### 3.3.1. Inhibitors of Viral Replication

**Figure 2:**
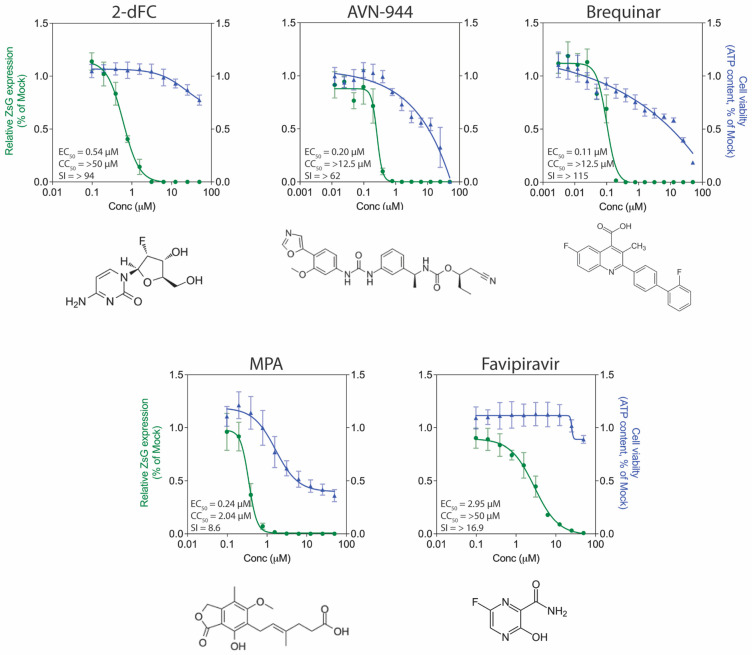
Antiviral activity of select compounds targeting viral replication processes. Concentration-response curves in Huh7 cells infected with rLUJV/ZsG (MOI 0.1) after being treated 1 h prior with 2′-deoxy-2′-fluorocytidine (2-dFC), AVN-944, brequinar, mycophenolic acid (MPA), or T-705. Relative ZsG expression is shown in green and cell viability in blue. Values were normalized to mock-treated (DMSO only) infected cells. Each point represents the mean of quadruplicate wells, with error bars indicating standard deviation; graphs are representative of at least 3 independent experiments. Associated EC_50_, CC_50_, and SI values are given, along with the chemical structure of each compound.

Some of the most potent anti-LUJV compounds and those with the highest SI in our screens were compounds predicted to target viral replication (Figure 2). The nucleoside analogs 2′-deoxy-2′-fluorocytidine (2-dFC) and favipiravir both had CC_50_ values > 50 µM, and EC_50_ values of 0.54 ± 0.21 µM and 2.95 ± 0.69 µM, respectively. 2-dFC has shown similar potency against LASV [16], the nairovirus Crimean-Congo hemorrhagic fever virus (CCHFV) [19], and the paramyxovirus Sosuga virus [21] in previous studies, further demonstrating this compound’s broad-species antiviral potential. The inosine-5′-monophosphate dehydrogenase inhibitors AVN-944 and mycophenolic acid (MPA) both demonstrated sub-micromolar EC_50_ values of 0.20 ± 0.09 µM and 0.24 ± 0.1 µM, respectively, although MPA’s CC_50_ value of 2.0 ± 0.4 µM resulted in a reduced SI of 8.6. MPA has previously demonstrated antiviral effects against a wide range of RNA viruses including influenza [22], dengue virus [23], Zika virus [24], rotavirus [25], CCHFV [19], and hantavirus [26]. AVN-944 was previously shown to have antiviral activity in vitro against JUNV, reducing titers by >90% at concentrations of 7.5 µM and above [27]. The best overall performing compound was brequinar (EC_50_ 0.109 ± 0.02 µM, CC_50_ > 12.5 µM, SI > 115), a selective inhibitor of the enzyme dihydroorotate dehydrogenase. This compound is believed to act by blocking de novo pyrimidine biosynthesis, and recent studies have shown brequinar inhibits other arenaviruses such as LASV, JUNV, and LCMV [28], as well as other RNA virus species such as influenza strains A and B [29] and HIV-1 [30].

#### 3.3.2. Inhibitors of Viral Entry, Trafficking, and Egress

**Figure 3:**
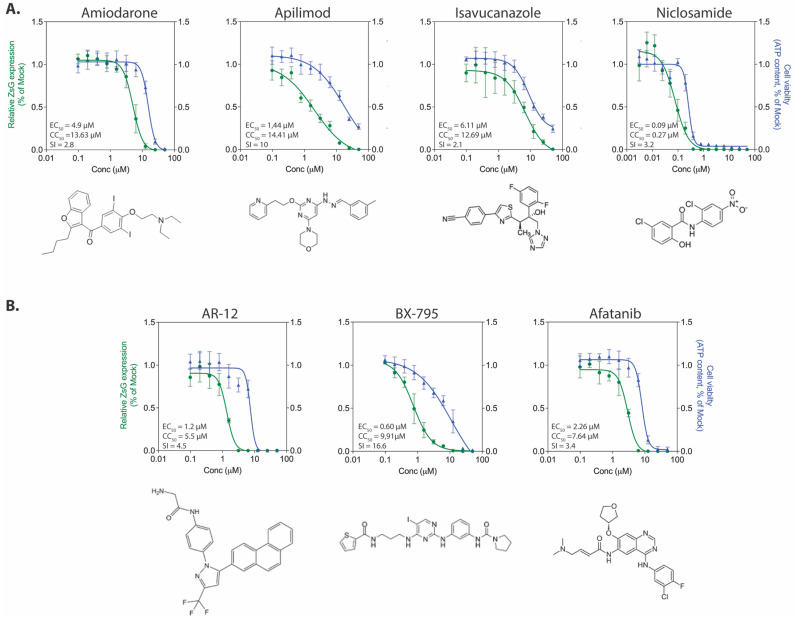
Antiviral activity of select protein kinase inhibitors and known entry inhibitors. Concentration-response curves in Huh7 cells infected with rLUJV/ZsG (MOI 0.1) after being treated 1 h prior with (**A**) compounds known to block arenavirus entry, egress, or intracellular tracking processes; or (**B**) protein kinase inhibitors. Relative ZsG expression is shown in green and cell viability is shown in blue. Values were normalized to mock-treated (DMSO only) infected cells. Each point represents the mean of quadruplicate wells, with error bars indicating standard deviation; graphs are representative of at least 3 independent experiments. Associated EC_50_, CC_50_, and SI values are provided along with the chemical structure of each compound.

The compounds apilimod, niclosamide, and isavucanazole have all been shown to inhibit LASV entry in vitro [31,32]. Of these, the most potent inhibitor of rLUJV/ZsG was niclosamide (EC_50_ 0.085 ± 0.029 µM, CC_50_ 0.272 ± 0.034 µM, SI = 3.2) with a sub-micromolar EC_50_ value (Figure 3A). Of the other two compounds, apilimod (EC_50_ 1.44 ± 0.6 µM, CC_50_ 14.41 ± 3.2 µM, SI = 10) demonstrated greater inhibition than isavucanazole (EC_50_ 6.1 ± 0.9 µM, CC_50_ 12.69 ± 3.9 µM, SI = 2.1). Interestingly, the niclosamide values for rLUJV/ZsG inhibition are very similar to the reported EC_50_ of 0.08 µM for LASV inhibition in a pseudovirus screening assay; however, the same study reported an EC_50_ of 0.05 µM for apilimod against LASV, which is considerably lower than EC = 1.44 µM for rLUJV/ZsG [31]. Isavucanazole was shown to inhibit LASV (EC_50_ 1.2 µM, CC_50_ > 30 µM) in an HIV-luc pseudovirus screen, but did not inhibit LUJV in the same screen [32]. Here, however, using rLUJV/ZsG, which better represents authentic viral replication, we observed a degree of inhibition using isavucanazole, which may indicate a mode of action different from blocking viral entry. Finally, amiodarone (EC_50_ 4.90 ± 0.51 µM, CC_50_ 13.63 ± 1.51 µM, SI = 2.8) is a cationic amphiphilic drug (CAD) that can accumulate within acidic intracellular vesicles, inhibiting membrane fusion and ribonucleoprotein release [33]. It can inhibit Ebola virus (EBOV) in vitro and in vivo [34,35], and has been shown to inhibit in vitro the new world arenavirus GTOV [36], SARS-CoV-1 [37], and hepatitis C virus [38]. While these four compounds all inhibited rLUJV/ZsG, the degree of cytotoxicity associated with all except apilimod reduced their respective SI values and resulted in a very narrow window of effectiveness.

#### 3.3.3. Kinase Inhibitors

Kinase inhibitors are increasingly being examined as potential antiviral drugs. They make attractive options for study since large numbers of these compounds are continually being developed and approved for clinical use to treat cancers and inflammatory conditions, and because of increasing knowledge of host kinase use by viruses during infection [39,40,41]. AR-12 is a celecoxib derivative kinase inhibitor that downregulates the PI3K/Akt pathway, which is known to contribute to arenavirus budding [42]. BX-795 inhibits 3-phosphoinositide-dependent kinase 1, IKK-related kinase, TANK-binding kinase 1, and IKKε; afatanib is a tyrosine kinase inhibitor. AR-12 has previously been shown to inhibit both LASV [18] and JUNV [43], and BX-795 has shown promise as a potent inhibitor of herpes simplex virus 1 (HSV-1) and HSV-2 [44]. Against rLUJV/ZsG, BX-795 (EC_50_ 0.60 ± 0.27 µM, CC_50_ 9.91 ± 1.09 µM) performed the best, with an SI value of 16.6. AR-12 (EC_50_ 1.3 ± 0.4 µM, CC_50_ 5.5 ± 1.7 µM, SI = 4.5) and afatinib (EC_50_ 2.26 ± 0.73 µM, CC_50_ 7.64 ± 0.7 µM, SI = 3.4) both inhibited rLUJV/ZsG, but their cytotoxicity reduced their SI values compared to BX-795 (Figure 3B).

#### 3.3.4. Selective Estrogen Receptor Modulators

**Figure 4:**
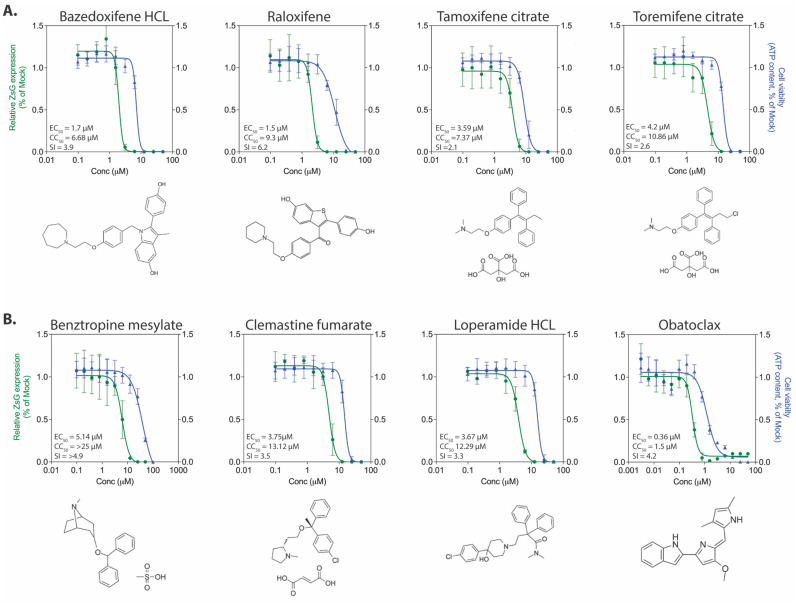
Antiviral activity of selective estrogen receptor modulators and additional compounds demonstrating antiviral effects. Concentration-response curves in Huh7 cells infected with rLUJV/ZsG (MOI 0.1) after being treated 1 h prior with: (**A**) selective estrogen receptor modulators (SERMs), or (**B**) protein kinase inhibitors. Relative ZsG expression is shown in green and cell viability is shown in blue. Values were normalized to mock-treated (DMSO only) infected cells. Each point represents the mean of quadruplicate wells, with error bars indicating standard deviation; graphs are representative of at least 3 independent experiments. Associated EC_50_, CC_50_, and SI values are provided, along with the chemical structure of each compound.

Four of the compounds in the screen were SERMs, a class of compounds that are also CADs and have antiviral effects against several viral species, including EBOV [45]. The SERM performing best against rLUJV/ZsG was raloxifene (EC_50_ 1.5 ± 0.79 µM, CC_50_ 9.3 ± 1.3 µM, SI = 6.2) (Figure 4A). Bazedoxifene HCl (EC_50_ 1.72 ± 0.79 µM, CC_50_ 6.68 ± 2.08 µM, SI = 3.9) demonstrated a similar EC_50_ value, but was associated with significant cytotoxicity, which reduced the SI values. Both tamoxifene citrate (EC_50_ 3.59 ± 0.72 µM, CC_50_ 7.37 ± 1.04 µM, SI = 2.1) and toremifene citrate (EC_50_ 4.22 ± 0.78 µM, CC_50_ 10.86 ± 3.14 µM, SI = 2.6) inhibited rLUJV/ZsG, but the associated cytotoxicity again led to a very narrow window of antiviral effect.

#### 3.3.5. Additional Compounds with Anti-rLUJV/ZsG Properties

Four of the compounds selected from the initial screen did not fit into the four previous groups based on predicted mode of action (Figure 4B). Three of these compounds are FDA-approved compounds: benztropine mesylate (EC_50_ 5.14 ± 1.52 µM, CC_50_ > 25, SI > 4.9) is a neurotransmitter inhibitor (anticholinergic); clemastine fumarate (EC_50_ 3.75 ± 1.79 µM, CC_50_ 13.12 ± 2.33 µM, SI = 3.5) acts primarily as an H1 histamine antagonist; and loperamide HCl (EC_50_ 3.67 ± 0.99 µM, CC_50_ 12.29 ± 3.57 µM, SI = 3.3) is an opioid receptor that targets μ-opioid receptors in the large intestine and acts as an antidiarrheal agent by decreasing intestinal movement. All three were able to moderately inhibit rLUJV/ZsG and had SI values between 3 and 5. The final compound, obatoclax (EC_50_ 0.36 ± 0.1 µM, CC_50_ 1.5 ± 0.33 µM, SI = 4.2) inhibits the Bcl-2 family of proteins and has been investigated as an experimental anti-cancer drug. It inhibited rLUJV/ZsG at sub-micromolar EC_50_ value, but was cytotoxic at a relatively low concentrations, which reduced SI to 4.2.

### 3.4. Confirmatory Screening with wt LUJV

**Figure 5:**
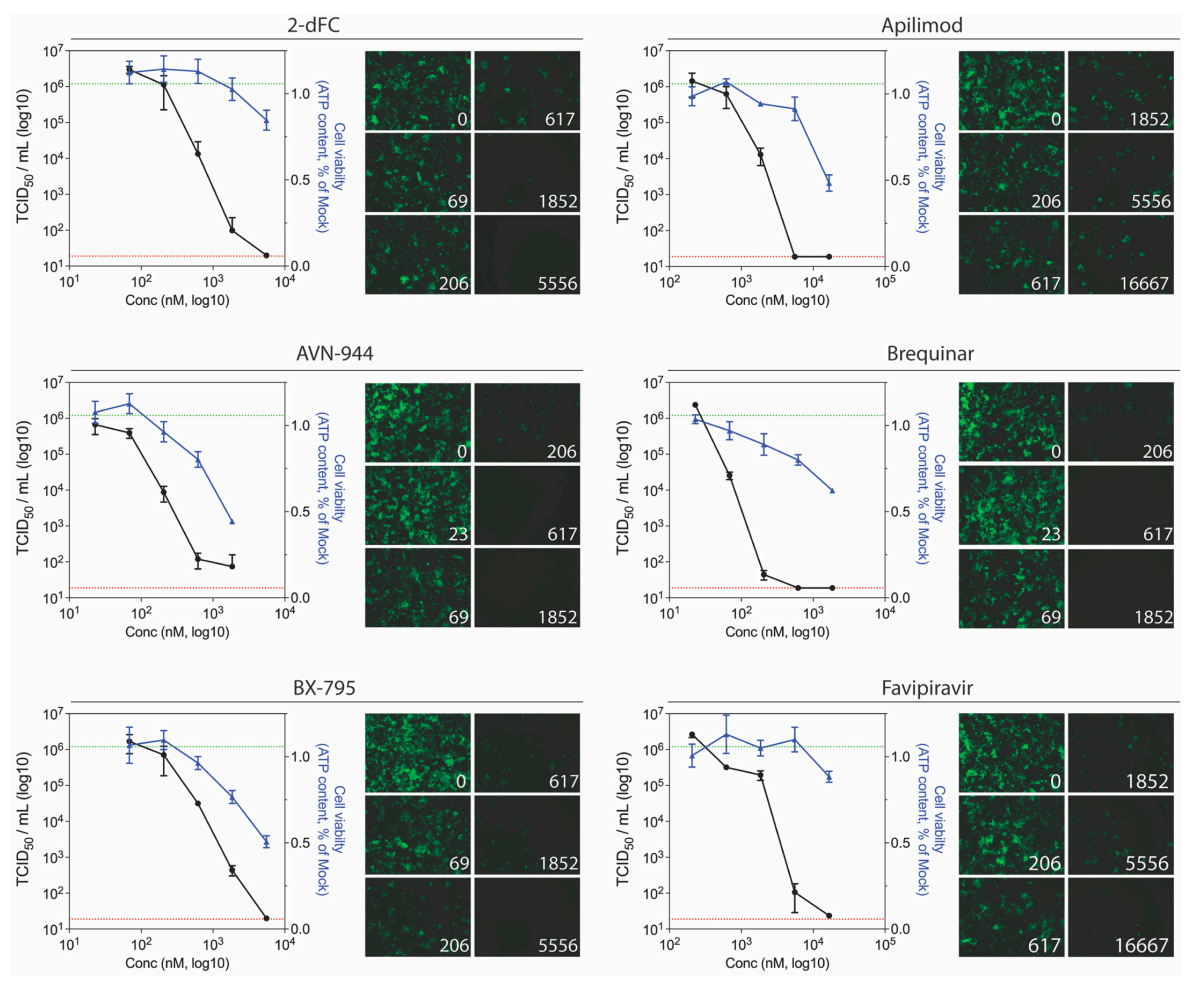
Ability of the most potent anti-LUJV compounds to inhibit wild-type virus replication. Titer reduction assays using wt LUJV were performed in Huh7 cells treated with varying concentrations of indicated compound 1 h prior to infection (MOI 0.1). For each compound concentration, inhibition of rLUJV/ZsG and cell viability were concurrently assessed. Four days post-infection, cell culture supernatants were collected and LUJV titers (TCID_50_/mL) were determined in Vero-E6 cells by immunofluorescence assays. For each concentration, LUJV titers (black) and cell viability (blue) are shown. Dotted lines represent the limit of detection for the TCID_50_ assay (red) and the mean wt LUJV titer in mock-treated (DMSO only) cells (green). Each point represents the mean of quadruplicate wells, with error bars indicating standard deviation; graphs are representative of at least 3 independent experiments. ZsG fluorescence in rLUJV/ZsG-infected Huh7 cells at each compound concentration is also shown (10× magnification).

To confirm that the antiviral activity of the compounds was not specific to the ZsG-expressing recombinant virus, the top six candidates (SI ≥ 10) were selected to be screened using wt LUJV; these compounds were 2-dFC, apilimod, AVN-944, brequinar, BX-795, and favipiravir. These six drugs were tested using a shortened concentration curve (5 dilutions centered around their calculated EC_50_ value), with cell viability determined concurrently. All compounds evaluated inhibited wt LUJV titers in a concentration-dependent manner, and all compounds reduced viral titers by approximately 4 logs while retaining >75% cell viability (Figure 5). These results further confirm the suitability of using a reduction of ZsG fluorescence in rLUJV/ZsG-infected cells as a convenient readout in place of wt virus infection.

## 4. Discussion

Only one outbreak of LUJV has been recorded to date, with four out of five patients succumbing to fatal disease. The effectiveness of existing antivirals in treating LUJV patients is unknown. Ribavirin was administered to the sole surviving patient; however, based on this single treated case, it is not possible to determine the effect of treatment on the outcome. Furthermore, given our knowledge of ribavirin treatment in other arenavirus infections, in which it is only partially effective and associated with significant side effects, investigations into uncovering effective and safe arenavirus therapeutic options are badly needed [7]. High-throughput screening assays utilizing recombinant reporter viruses as surrogates for the wild-type parental virus have previously been successful in identifying several anti-mammarenavirus compounds [15,16,46,47]. Here, using a novel recombinant reporter LUJV, we were able to identify six compounds with high efficacy against LUJV that warrant further evaluation.

Given the genetic diversity among species, the large number of different arenavirus host reservoirs, and the geographic range of these pathogens, the emergence of new arenaviruses pathogenic to humans remains an ongoing public health concern [7]. Importantly, several of the compounds identified here as effective against LUJV (i.e., favipiravir, 2-dFC, and brequinar) have also been shown to inhibit other arenavirus species. The nucleoside analogs favipiravir and 2-dFC are both known to inhibit other arenaviruses including LASV, JUNV, Machupo virus, and GTOV [16,48,49]. The most promising inhibitor identified here was the dihydroorotate dehydrogenase inhibitor brequinar (EC_50_ 0.11 ± 0.02 µM, CC_50_ > 12.5 µM, SI > 115), which has previously been shown to inhibit LASV, JUNV, and LCMV at similar sub-micromolar EC_50_ values [50]. These broad-spectrum antivirals inhibit a wide range of highly pathogenic arenavirus species, presumably by targeting conserved viral processes, and are thus promising candidates for treating current and future emerging arenaviruses. It should be noted that inhibitors of pyrimidine biosynthesis can exhibit potent antiviral activity in cell culture, but be ineffective in animal models, which is likely due to pyrimidines in the animals’ diet overcoming the antiviral effect [51].

There is notable value in identifying efficacious compounds that have previously been approved for clinical use. Several of the compounds with anti-LUJV efficacy are already approved for other indications. Benztropine (Congentin) is used as an anti-tremor medication to treat Parkinson’s disease, and has also been shown to have mild antiviral effects against EBOV (EC_50_ 8.1 µM) [52], MERS-CoV (EC_50_ 16.6 µM), and SARS-CoV (EC_50_ 21.6 µM) [53]. Clemastine, an approved anti-histamine, is also effective against EBOV (EC_50_ 5.4 µM) [52], SARS-CoV-2 (EC_50_ 0.95 µM), and SARS-CoV-1 (EC_50_ 6.6 ± 1.48 µM) [54]. While not yet approved, obatoclax is an inhibitor of the Bcl-2 family of proteins and an experimental anti-cancer drug that has undergone several phase II clinical trials; it has been shown to inhibit LCMV [28] as well as multiple other virus species including Rift Valley fever virus and HSV-2 [30]. The ability to rapidly screen large libraries of FDA-approved compounds or small molecules for antiviral efficacy is a huge advantage of our reporter virus systems, since comparable studies using wt virus are difficult to adapt for high-throughput screening and are prohibitively time-consuming, costly, and labor-intensive.

The six most promising candidates with both high efficacy and low cytotoxicity fell within three broadly defined groups based on modes of action: inhibiting viral replication; viral entry and trafficking; or protein kinases. The majority (four out of six) of these with the highest SI values (and therefore the most promising therapeutic candidates) were inhibitors of viral replication. Several other compounds that were identified demonstrated potent anti-LUJV activity, but had associated cytotoxicity, leading to low SI values and a narrow therapeutic window. Although this would likely make these compounds unattractive as single-drug treatment options, combination therapy using compounds with distinct modes of action may synergistically inhibit LUJV [19,27,55,56]. This would allow these more cytotoxic compounds to be used at far lower concentrations while maximizing their antiviral potential.

Here we describe the development of a recombinant LUJV engineered to express the fluorescent protein ZsGreen1 and validate its use in high-throughput screening assays for large or complex screens containing multiple compounds at various concentrations. We identified several highly efficacious anti-LUJV compounds with multiple predicted modes of action. Future studies are planned to investigate if potential synergistic actions increase the effectiveness of these compounds, as well as to advance the most promising candidates to studies using the available animal models of disease to evaluate in vivo effectiveness. Given the genetic diversity of mammarenaviruses and the high-consequence nature of their infections, it is promising to report that newly emerging species such as LUJV are similarly inhibited by several previously reported compounds. Identifying pan-arenavirus inhibiting compounds serves as a key step in the preparedness against new and emerging pathogenic threats.

## Figures and Tables

**Table 1:** 50% effective concentration (EC_50_), 50% cytotoxic concentration (CC_50_), and selectivity indices (SI) of select compounds identified by rLUJV/ZsG reporter virus screening assay.

Inhibition Group	Compound	EC_50_ (µM)	CC_50_ (µM)	SI
Viral replication	2′-deoxy-2′-fluorocytidine (2-dFC)	0.533 ± 0.205	>50	>94
	AVN-944	0.201 ± 0.089	>12.5	>62
	Brequinar	0.109 ± 0.015	>12.5	>115
	Mycophenolic acid (MPA)	0.238 ± 0.097	2.035 ± 0.380	8.6
	Favipiravir (T-705)	2.951 ± 0.693	>50	>16.9
Viral entry, uncoating, and egress	Amiodarone	4.896 ± 0.513	13.63 ± 1.514	2.8
	Apilimod	1.438 ± 0.637	14.41 ± 3.245	10
	Isavucanazole	6.107 ± 0.926	12.69 ± 3.970	2.1
	Niclosamide	0.085 ± 0.029	0.272 ± 0.034	3.2
Protein kinase	AR-12	1.225 ± 0.444	5.522 ± 1.670	4.5
	BX-795	0.598 ± 0.274	9.913 ± 1.091	16.6
	Afatinib	2.256 ± 0.734	7.639 ± 0.699	3.4
SERMs	Bazedoxifene HCL	1.719 ± 0.481	6.679 ± 2.077	3.9
	Raloxifene (Evista)	1.500 ± 0.793	9.295 ± 1.314	6.2
	Tamoxifene citrate	3.594 ± 0.724	7.371 ± 1.036	2.1
	Toremifene citrate	4.217 ± 0.778	10.86 ± 3.144	2.6
Additional mechanisms	Benztropine mesylate	5.141 ± 1.515	>25	>4.9
	Clemastine fumarate	3.750 ± 1.790	13.12 ± 2.328	3.5
	Loperamide HCL	3.669 ± 0.986	12.29 ± 3.574	3.3
	Obatoclax	0.355 ± 0.105	1.502 ± 0.332	4.2

## Data Availability

Not applicable.

## Supplementary Materials

The following are available online at https://www.mdpi.com/article/10.3390/v13071255/s1. Table S1: Screening a panel of selected compounds for inhibition of rLUJV/ZsG fluorescence.

## References

[1] Paweska J.T., Sewlall N.H., Ksiazek T.G., Blumberg L.H., Hale M.J., Lipkin W.I., Weyer J., Nichol S.T., Rollin P.E., McMullan L.K. (2009). Nosocomial outbreak of novel arenavirus infection, southern Africa. Emerg. Infect. Dis..

[2] Briese T., Paweska J.T., McMullan L.K., Hutchison S.K., Street C., Palacios G., Khristova M.L., Weyer J., Swanepoel R., Egholm M. (2009). Genetic detection and characterization of Lujo virus, a new hemorrhagic fever-associated arenavirus from southern Africa. PLoS Pathog..

[3] Radoshitzky S.R., Buchmeier M.J., Charrel R.N., Clegg J.C.S., Gonzalez J.-P.J., Günther S., Hepojoki J., Kuhn J.H., Lukashevich I.S., Romanowski V. (2019). ICTV Virus Taxonomy Profile: Arenaviridae. J. Gen. Virol..

[4] Gonzalez J.P., Emonet S., de Lamballerie X., Charrel R. (2007). Arenaviruses. Curr. Top. Microbiol. Immunol..

[5] Hallam S.J., Koma T., Maruyama J., Paessler S. (2018). Review of Mammarenavirus Biology and Replication. Front. Microbiol..

[6] Charrel R.N., de Lamballerie X. (2010). Zoonotic aspects of arenavirus infections. Veter. Microbiol..

[7] Emonet S.F., de la Torre J.C., Domingo E., Sevilla N. (2009). Arenavirus genetic diversity and its biological implications. Infect. Genet. Evol..

[8] Radoshitzky S.R., Bào Y., Buchmeier M.J., Charrel R.N., Clawson A.N., Clegg C.S., DeRisi J.L., Emonet S., Gonzalez J.-P., Kuhn J.H. (2015). Past, present, and future of arenavirus taxonomy. Arch. Virol..

[9] Maiztegui J.I., Fernandez N.J., de Damilano A.J. (1979). Efficacy of immune plasma in treatment of Argentine haemorrhagic fever and association between treatment and a late neurological syndrome. Lancet.

[10] Raabe V.N., Kann G., Ribner B.S., Morales A., Varkey J.B., Mehta A.K., Lyon G.M., Vanairsdale S., Faber K., Becker S. (2017). Favipiravir and Ribavirin Treatment of Epidemiologically Linked Cases of Lassa Fever. Clin. Infect. Dis..

[11] Brisse M.E., Ly H. (2019). Hemorrhagic Fever-Causing Arenaviruses: Lethal Pathogens and Potent Immune Suppressors. Front. Immunol..

[12] Bergeron É., Chakrabarti A.K., Bird B.H., Dodd K.A., McMullan L.K., Spiropoulou C.F., Nichol S.T., Albariño C.G. (2012). Reverse genetics recovery of Lujo virus and role of virus RNA secondary structures in efficient virus growth. J. Virol..

[13] Reed L.J., Muench H. (1938). A simple method for estimating fifty percent endpoints. Am. J. Epidemiol..

[14] Emonet S.F., Garidou L., McGavern D.B., de la Torre J.C. (2009). Generation of recombinant lymphocytic choriomeningitis viruses with trisegmented genomes stably expressing two additional genes of interest. Proc. Natl. Acad. Sci. USA.

[15] Caì Y., Iwasaki M., Beitzel B.F., Yú S., Postnikova E.N., Cubitt B., DeWald L.E., Radoshitzky S.R., Bollinger L., Jahrling P.B. (2018). Recombinant Lassa Virus Expressing Green Fluorescent Protein as a Tool for High-Throughput Drug Screens and Neutralizing Antibody Assays. Viruses.

[16] Welch S.R., Guerrero L.W., Chakrabarti A.K., McMullan L.K., Flint M., Bluemling G.R., Painter G.R., Nichol S.T., Spiropoulou C.F., Albariño C.G. (2016). Lassa and Ebola virus inhibitors identified using minigenome and recombinant virus reporter systems. Antivir. Res..

[17] Kim J.H., Lee S.R., Li L.H., Park H.J., Park J.H., Lee K.Y., Kim M.K., Shin B.A., Choi S.Y. (2011). High cleavage efficiency of a 2A peptide derived from porcine teschovirus-1 in human cell lines, zebrafish and mice. PLoS ONE.

[18] Mohr E.L., McMullan L.K., Lo M.K., Spengler J.R., Bergeron É., Albariño C.G., Shrivastava-Ranjan P., Chiang C.-F.F., Nichol S.T., Spiropoulou C.F. (2015). Inhibitors of cellular kinases with broad-spectrum antiviral activity for hemorrhagic fever viruses. Antivir. Res..

[19] Welch S.R., Scholte F.E.M.M., Flint M., Chatterjee P., Nichol S.T., Bergeron É., Spiropoulou C.F. (2017). Identification of 2’-deoxy-2’-fluorocytidine as a potent inhibitor of Crimean-Congo hemorrhagic fever virus replication using a recombinant fluorescent reporter virus. Antivir. Res..

[20] García C.C., Candurra N.A., Damonte E.B. (2000). Antiviral and virucidal activities against arenaviruses of zinc-finger active compounds. Antivir. Chem. Chemother..

[21] Welch S.R., Chakrabarti A.K., Wiggleton Guerrero L., Jenks H.M., Lo M.K., Nichol S.T., Spiropoulou C.F., Albariño C.G. (2018). Development of a reverse genetics system for Sosuga virus allows rapid screening of antiviral compounds. PLoS Negl. Trop. Dis..

[22] To K.K.W., Mok K.-Y., Chan A.S.F., Cheung N.N., Wang P., Lui Y.-M., Chan J.F.W., Chen H., Chan K.-H., Kao R.Y.T. (2016). Mycophenolic acid, an immunomodulator, has potent and broad-spectrum in vitro antiviral activity against pandemic, seasonal and avian influenza viruses affecting humans. J. Gen. Virol..

[23] Diamond M.S., Zachariah M., Harris E. (2002). Mycophenolic Acid Inhibits Dengue Virus Infection by Preventing Replication of Viral RNA. Virology.

[24] Barrows N.J., Campos R.K., Powell S.T., Prasanth K.R., Schott-Lerner G., Soto-Acosta R., Galarza-Muñoz G., McGrath E.L., Urrabaz-Garza R., Gao J. (2016). A Screen of FDA-Approved Drugs for Inhibitors of Zika Virus Infection. Cell Host Microbe.

[25] Yin Y., Wang Y., Dang W., Xu L., Su J., Zhou X., Wang W., Felczak K., van der Laan L.J.W., Pankiewicz K.W. (2016). Mycophenolic acid potently inhibits rotavirus infection with a high barrier to resistance development. Antivir. Res..

[26] Sun Y., Chung D.-H., Chu Y.-K., Jonsson C.B., Parker W.B. (2007). Activity of ribavirin against Hantaan virus correlates with production of ribavirin-5’-triphosphate, not with inhibition of IMP dehydrogenase. Antimicrob. Agents Chemother..

[27] Dunham E.C., Leske A., Shifflett K., Watt A., Feldmann H., Hoenen T., Groseth A. (2018). Lifecycle modelling systems support inosine monophosphate dehydrogenase (IMPDH) as a pro-viral factor and antiviral target for New World arenaviruses. Antivir. Res..

[28] Kim Y.-J., Cubitt B., Chen E., Hull M.V., Chatterjee A.K., Cai Y., Kuhn J.H., de la Torre J.C. (2019). The ReFRAME library as a comprehensive drug repurposing library to identify mammarenavirus inhibitors. Antivir. Res..

[29] Park J.-G., Ávila-Pérez G., Nogales A., Blanco-Lobo P., de la Torre J.C., Martínez-Sobrido L. (2020). Identification and Characterization of Novel Compounds with Broad-Spectrum Antiviral Activity against Influenza A and B Viruses. J. Virol..

[30] Andersen P.I., Krpina K., Ianevski A., Shtaida N., Jo E., Yang J., Koit S., Tenson T., Hukkanen V., Anthonsen M.W. (2019). Novel Antiviral Activities of Obatoclax, Emetine, Niclosamide, Brequinar, and Homoharringtonine. Viruses.

[31] Hulseberg C.E., Fénéant L., Szymańska-de Wijs K.M., Kessler N.P., Nelson E.A., Shoemaker C.J., Schmaljohn C.S., Polyak S.J., White J.M. (2019). Arbidol and Other Low-Molecular-Weight Drugs That Inhibit Lassa and Ebola Viruses. J. Virol..

[32] Zhang X., Tang K., Guo Y. (2020). The antifungal isavuconazole inhibits the entry of lassa virus by targeting the stable signal peptide-GP2 subunit interface of lassa virus glycoprotein. Antivir. Res..

[33] Salata C., Calistri A., Parolin C., Baritussio A., Palù G. (2017). Antiviral activity of cationic amphiphilic drugs. Expert Rev. Anti. Infect. Ther..

[34] Salata C., Baritussio A., Munegato D., Calistri A., Ha H.R., Bigler L., Fabris F., Parolin C., Palù G., Mirazimi A. (2015). Amiodarone and metabolite MDEA inhibit Ebola virus infection by interfering with the viral entry process. Pathog. Dis..

[35] Madrid P.B., Panchal R.G., Warren T.K., Shurtleff A.C., Endsley A.N., Green C.E., Kolokoltsov A., Davey R., Manger I.D., Gilfillan L. (2015). Evaluation of Ebola Virus Inhibitors for Drug Repurposing. ACS Infect. Dis..

[36] Gehring G., Rohrmann K., Atenchong N., Mittler E., Becker S., Dahlmann F., Pöhlmann S., Vondran F.W.R., David S., Manns M.P. (2014). The clinically approved drugs amiodarone, dronedarone and verapamil inhibit filovirus cell entry. J. Antimicrob. Chemother..

[37] Stadler K., Ha H.R., Ciminale V., Spirli C., Saletti G., Schiavon M., Bruttomesso D., Bigler L., Follath F., Pettenazzo A. (2008). Amiodarone alters late endosomes and inhibits SARS coronavirus infection at a post-endosomal level. Am. J. Respir. Cell Mol. Biol..

[38] Cheng Y.-L., Lan K.-H., Lee W.-P., Tseng S.-H., Hung L.-R., Lin H.-C., Lee F.-Y., Lee S.-D., Lan K.-H. (2013). Amiodarone inhibits the entry and assembly steps of hepatitis C virus life cycle. Clin. Sci..

[39] Schor S., Einav S. (2018). Repurposing of Kinase Inhibitors as Broad-Spectrum Antiviral Drugs. DNA Cell Biol..

[40] Ott P.A., Adams S. (2011). Small-molecule protein kinase inhibitors and their effects on the immune system: Implications for cancer treatment. Immunotherapy.

[41] Gross S., Rahal R., Stransky N., Lengauer C., Hoeflich K.P. (2015). Targeting cancer with kinase inhibitors. J. Clin. Investig..

[42] Urata S., Ngo N., de la Torre J.C. (2012). The PI3K/Akt pathway contributes to arenavirus budding. J. Virol..

[43] Booth L., Roberts J.L., Ecroyd H., Tritsch S.R., Bavari S., Reid S.P., Proniuk S., Zukiwski A., Jacob A., Sepúlveda C.S. (2016). AR-12 Inhibits Multiple Chaperones Concomitant With Stimulating Autophagosome Formation Collectively Preventing Virus Replication. J. Cell. Physiol..

[44] Su A.-R., Qiu M., Li Y.-L., Xu W.-T., Song S.-W., Wang X.-H., Song H.-Y., Zheng N., Wu Z.-W. (2017). BX-795 inhibits HSV-1 and HSV-2 replication by blocking the JNK/p38 pathways without interfering with PDK1 activity in host cells. Acta Pharmacol. Sin..

[45] Johansen L.M., Brannan J.M., Delos S.E., Shoemaker C.J., Stossel A., Lear C., Hoffstrom B.G., Dewald L.E., Schornberg K.L., Scully C. (2013). FDA-approved selective estrogen receptor modulators inhibit Ebola virus infection. Sci. Transl. Med..

[46] Lee A.M., Rojek J.M., Gundersen A., Ströher U., Juteau J.-M., Vaillant A., Kunz S. (2008). Inhibition of cellular entry of lymphocytic choriomeningitis virus by amphipathic DNA polymers. Virology.

[47] Herring S., Oda J.M., Wagoner J., Kirchmeier D., O’Connor A., Nelson E.A., Huang Q., Liang Y., DeWald L.E., Johansen L.M. (2021). Inhibition of Arenaviruses by Combinations of Orally Available Approved Drugs. Antimicrob. Agents Chemother..

[48] Gowen B.B., Juelich T.L., Sefing E.J., Brasel T., Smith J.K., Zhang L., Tigabu B., Hill T.E., Yun T., Pietzsch C. (2013). Favipiravir (T-705) inhibits Junín virus infection and reduces mortality in a guinea pig model of Argentine hemorrhagic fever. PLoS Negl. Trop. Dis..

[49] Mendenhall M., Russell A., Juelich T., Messina E.L., Smee D.F., Freiberg A.N., Holbrook M.R., Furuta Y., de la Torre J.-C., Nunberg J.H. (2011). T-705 (favipiravir) inhibition of arenavirus replication in cell culture. Antimicrob. Agents Chemother..

[50] Kim Y.-J., Cubitt B., Cai Y., Kuhn J.H., Vitt D., Kohlhof H., de la Torre J.C. (2020). Novel Dihydroorotate Dehydrogenase Inhibitors with Potent Interferon-Independent Antiviral Activity against Mammarenaviruses In Vitro. Viruses.

[51] Wang Q.-Y., Bushell S., Qing M., Xu H.Y., Bonavia A., Nunes S., Zhou J., Poh M.K., Florez de Sessions P., Niyomrattanakit P. (2011). Inhibition of dengue virus through suppression of host pyrimidine biosynthesis. J. Virol..

[52] Johansen L.M., DeWald L.E., Shoemaker C.J., Hoffstrom B.G., Lear-Rooney C.M., Stossel A., Nelson E., Delos S.E., Simmons J.A., Grenier J.M. (2015). A screen of approved drugs and molecular probes identifies therapeutics with anti-Ebola virus activity. Sci. Transl. Med..

[53] Dyall J., Coleman C.M., Hart B.J., Venkataraman T., Holbrook M.R., Kindrachuk J., Johnson R.F., Olinger G.G., Jahrling P.B., Laidlaw M. (2014). Repurposing of clinically developed drugs for treatment of Middle East respiratory syndrome coronavirus infection. Antimicrob. Agents Chemother..

[54] Yang L., Pei R., Li H., Ma X., Zhou Y., Zhu F., He P., Tang W., Zhang Y., Xiong J. (2020). Identification of SARS-CoV-2 entry inhibitors among already approved drugs. Acta Pharmacol. Sin..

[55] Delang L., Abdelnabi R., Neyts J. (2018). Favipiravir as a potential countermeasure against neglected and emerging RNA viruses. Antivir. Res..

[56] Pomeroy J.J., Drusano G.L., Rodriquez J.L., Brown A.N. (2017). Searching for synergy: Identifying optimal antiviral combination therapy using Hepatitis C virus (HCV) agents in a replicon system. Antivir. Res..

