# Supplementary material for: Screening and Identification of Lujo Virus Inhibitors Using a Recombinant Reporter Virus Platform
Source: Viruses. 2021 Jun 28;13(7):1255. doi: 10.3390/v13071255 (PMC8310135; doi:10.3390/v13071255)
Supplement: Supplementary file 1 [file viruses-13-01255-s001.zip › viruses-1263721-supplementary.pdf]

Supplementary Table 1. Screening a panel of selected compounds for inhibition of LUJV/ZsG fluorescence Compounds were screening for activity in Huh7 cells infected with LUJV/ZsG at MOI 0.1 one hour after treatment at concentrations of 5000, 500, and 50 nM. Cell viability was determined concurrently by assessing ATP content.

Compound Name	Relative ZsG (%)						Cell Viability (%)					
	5000 nM		500 nM		50 nM		5000 nM		500 nM		50 nM	
	Mean	SD	Mean	SD	Mean	SD	Mean	SD	Mean	SD	Mean	SD
2-chloroadenosine	99	24	133	4	124	15	103	2	105	5	98	3
2'-deoxy-2'-fluorocytidine	2	0	27	5	90	2	105	0	98	13	117	6
2'-C-methylcytidine	103	29	144	29	124	29	99	2	104	3	102	2
2'-C-methyluridine	126	3	142	24	132	13	97	6	100	5	102	4
2'-O-methylcytidine	114	28	127	27	118	6	100	6	103	1	98	7
2'-O-methyluridine	110	54	130	29	122	18	96	7	100	5	97	2
3-deazauridine	111	21	105	12	108	35	99	1	96	1	103	6
5-azacytidine	104	14	112	7	90	12	102	6	102	1	96	3
5-azidouridine	113	18	119	13	121	18	104	3	100	1	104	5
5-bromouridine	106	4	131	4	134	7	100	3	102	6	97	4
5-chlorouridine	103	17	142	6	122	11	99	2	103	3	100	3
5-fluorouridine	19	4	51	4	94	5	103	7	100	6	97	7
5-methylcytidine	108	3	129	11	122	27	98	7	97	3	96	5
6-aza-2-thiouridine	112	25	139	4	134	18	103	3	96	5	104	5
6-azauridine	78	8	87	27	95	7	97	7	103	5	98	5
7-DMA	92	5	95	26	96	4	113	7	103	2	98	1
8-azaadenosine	6	0	121	17	118	11	47	6	103	3	98	2
8-azidoadenosine	6	0	72	10	90	7	32	3	87	4	104	3
ABT-333	129	7	111	8	131	7	104	3	96	7	99	7
Adefovir dipivoxil	24	3	38	4	70	4	54	0	94	11	123	5
Afatinib	2	0	74	14	76	8	60	11	136	17	126	4
Amiodarone	3	1	60	9	80	8	86	13	145	3	132	1
Apatinib	47	7	67	15	91	40	95	10	119	11	113	4
Apilimod	24	4	45	4	91	10	80	6	115	5	130	3
AR-12 (OSU-03012)	2	0	54	7	74	21	1	0	115	4	129	4
Arbidol	40	9	65	21	77	9	109	7	139	11	135	7
Aripiprazole	16	6	81	5	71	12	59	7	126	2	124	7
Atovaquone	74	12	90	8	108	25	115	9	134	12	129	3
AVN-944	1	0	4	1	43	8	64	12	97	7	103	12
AZD8055	3	0	7	1	48	2	45	1	52	2	86	8
Balapiravir (R1626)	122	31	81	35	139	5	99	7	104	4	104	6
Bazedoxifene HCl	2	0	103	11	110	10	84	6	147	8	141	1
BCX-4430	99	7	132	17	103	14	101	5	97	7	99	5
Benidipine	61	0	81	14	90	9	107	10	129	11	126	2
Benztropine mesylate	36	3	90	5	110	23	108	15	130	5	126	3
Brequinar	2	0	2	0	27	14	67	5	85	7	113	4
BX-795	9	2	41	4	80	3	60	8	105	9	128	4
Clemastine fumarate	6	3	81	7	95	8	107	2	116	6	115	1
Clofazimine	52	3	75	16	88	3	102	10	125	14	120	6
Clomipramine HCL	12	8	103	9	129	19	98	6	97	7	97	2
Dabrafenib	22	2	79	17	87	28	68	9	117	12	119	4
Duloxetine HCL	4	2	74	13	103	12	76	12	109	14	104	15

Erlotinib	65	1	66	6	82	13	110	4	126	7	127	2
Evista (raloxifene)	3	1	89	20	87	8	86	5	136	11	139	2
Gefitinib	48	8	71	2	77	21	101	4	121	1	128	4
Genistein	92	10	68	4	83	13	106	0	111	13	123	8
Gleevec	56	18	113	11	95	4	96	4	96	5	101	4
Isavuconazole	34	8	75	10	73	9	75	5	121	3	114	4
Lacidipine	47	10	90	7	90	20	98	12	129	15	138	3
Gefitinib	48	8	71	2	77	21	101	4	121	1	128	4
Lamuvudine	102	16	117	15	77	11	104	6	97	7	95	5
Loperamide HCL	1	0	81	9	104	8	85	9	104	9	99	7
Losmapimod	63	5	70	2	60	6	114	6	115	3	113	3
Miconazole nitrate	47	1	78	5	65	16	67	2	120	4	119	7
Mycophenolic acid	2	0	7	1	72	5	48	4	100	7	132	2
Nebivolol	18	5	58	18	76	16	76	5	108	7	110	3
NH125	2	0	28	9	60	12	1	0	63	10	104	7
Niclosamide	2	0	2	0	30	6	1	0	2	0	77	4
NITD008	136	7	110	3	122	14	97	4	96	5	99	7
Obatoclax	10	1	4	0	36	7	2	1	25	4	130	1
Paroxetine HCl	48	4	83	6	93	12	110	1	145	6	138	0
Pazopanib HCl	24	5	59	4	77	17	54	3	116	4	134	1
PRT062607 HCl	8	6	70	15	89	34	79	8	114	5	113	3
PSI-6130	114	42	90	20	149	3	99	5	96	2	100	5
PSI-6206	128	36	87	12	141	17	104	1	96	5	103	5
PSI-7976	124	39	80	11	96	9	107	1	99	3	94	2
PSI-7977	118	16	95	7	119	12	104	6	97	5	101	3
R-1479	103	32	100	31	118	6	96	3	101	5	104	7
R-7128	126	28	80	20	133	23	103	2	98	2	100	7
R09187	88	12	122	16	116	6	102	4	98	4	99	6
Ribavirin	63	11	73	6	94	21	133	6	122	11	124	3
Sertraline	35	5	69	7	80	1	107	8	128	11	122	6
Stavudine	96	21	104	19	125	30	103	6	98	7	101	6
ST-193	91	12	102	5	107	11	101	7	98	6	105	8
Sulconazole nitrate	42	5	66	12	86	11	52	12	131	7	132	0
T-705	22	8	95	11	94	14	96	7	104	3	101	1
Tamoxifen citrate	2	0	78	20	84	4	85	5	123	8	123	3
Tenofivir	96	28	138	27	114	5	100	4	100	3	101	6
Tetrandrine	2	0	75	0	95	6	1	0	95	4	115	6
Toremifene citrate	9	2	88	7	86	26	93	7	128	12	122	0
Toyocamycin	7	0	12	1	69	5	23	4	64	9	97	2
TWS119	13	3	74	18	74	17	36	2	124	36	135	4
Tyrphostin AG1478	87	7	73	0	80	4	111	3	101	16	120	2
Zidovudine	92	9	88	10	99	10	93	9	105	2	97	4
